# Arabic version of the intermittent and constant osteoarthritis pain questionnaire (ICOAP-Ar): translation, cross-cultural adaptation, and measurement properties

**DOI:** 10.1186/s12891-023-06492-w

**Published:** 2023-06-13

**Authors:** Ahmed Farrag, Walaa Elsayed, Doaa Al Saleh, Ahmed Hefny, Afaf Shaheen

**Affiliations:** 1 Basic Science Department, Faculty of Physical Therapy, Cairo University, Cairo, Egypt; 2 Department of Physical Therapy, College of Applied Medical Sciences, Imam Abdulrahman Bin Faisal University, Dammam, Saudi Arabia; 3 Department of Physical Therapy, King Fahd Military Medical Complex, Dhahran, Saudi Arabia; 4 Therapy & Rehab. Center, Hurghada, Egypt; 5 Department of Health Rehabilitation Sciences, College of Applied Medical Sciences, King Saud University, Riyadh, Saudi Arabia

**Keywords:** Pain, Osteoarthritis, Knee, ICOAP

## Abstract

**Background:**

Pain is the most incapacitating symptom of knee osteoarthritis (OA), with intermittent and/or continuous nature as described by the patients. Accuracy of pain assessment tools across different cultures is important. This study aimed to translate and culturally adapt the Intermittent and Constant OsteoArthritis Pain (ICOAP) measure into Arabic (ICOAP-Ar) and evaluate its psychometric properties in patients with knee OA.

**Methods:**

The ICOAP was cross-culturally adapted following the recommended guidelines from English. Knee OA patients from outpatient clinics were recruited to assess the structural (confirmatory factor analysis) and construct validity (Spearman’s correlation coefficient - rho) to assess the relationship between the ICOAP-Ar and the pain and symptoms subscales of the Knee Injury and Osteoarthritis Outcome Score (KOOS), in addition to internal consistency (Cronbach’s alpha and the corrected item-total correlation). A week later, test-retest reliability (intraclass correlation coefficient (ICC)) was evaluated. Following four weeks of physical therapy treatment, the ICOAP-Ar responsiveness was evaluated using the receiver operating characteristic curve.

**Results:**

Ninety-seven participants were recruited (age = 52.97 ± 9.9). A model with single pain construct showed acceptable fit (Comparative fit index = 0.92). The ICOAP-Ar total and subscales had a strong to moderate negative correlation with the KOOS pain and symptoms domains, respectively. The ICOAP-Ar total and subscales demonstrated satisfactory internal consistency (α = 0.86–0.93). The ICCs were excellent (ICCs = 0.89–0.92) with acceptable corrected item total correlations (rho = 0.53–0.87) for the ICOAP-Ar items. The ICOAP-Ar responsiveness was good with moderate effect size (ES = 0.51–0.65) and large standardized response mean (SRM = 0.86–0.99). A cut-off point of 51.1/100 was determined with moderate accuracy (Area under the curve = 0.81, sensitivity = 85%, specificity = 71%). No floor or ceiling effects were found.

**Conclusions:**

The ICOAP-Ar exhibited good validity, reliability, and responsiveness after physical therapy treatment for knee OA, which renders it reliable for evaluating knee OA pain in clinical and research settings.

**Supplementary Information:**

The online version contains supplementary material available at 10.1186/s12891-023-06492-w.

## Introduction

Joint osteoarthritis (OA) is an active pathological joint condition associated with considerable multifactorial health burden [1, 2]. It is attributed to an unbalanced repair-damage process of variable joint structures [3]. It is commonly associated with structural changes and pro-inflammatory reactions [4, 5]. Amongst a multiplicity of symptoms, pain is the most incapacitating clinical symptom joint OA patients always report to clinicians [6]. Several sets of criteria have been developed to help clinically diagnose joint OA, and joint pain was the main diagnostic symptom in common [7–9]. This indicates the critical importance of joint pain assessment for diagnostic and prognostic purposes of joint OA.

Lower extremity joints are the most affected by OA. This made the lower extremity joints the most common joints requiring replacement surgeries [10, 11]. Knee OA comprises 85% of the OA global burden [12]. A year prevalence of knee OA was recently reported to reach 22.9%, corresponding to 654.1 million individuals aged 40 and above [13]. Typically, OA joint pain is intermittent and may occasionally flare-up with increased intensity and frequency and decreased threshold [14, 15]. Research has reported a significant relationship between the severity of knee OA pain and poor sleep quality and reduced quality of life [16, 17].

The availability of a valid tool for measuring knee OA pain is important for patient assessment and follow-up. The importance is greatly emphasized with the conduction of multicenter and multinational studies targeting patients with knee OA. This necessitates standardized measurement tools for producing comparable and meaningful data. This is achieved by cross-cultural adaptation of a measurement tool across different languages. Focus groups examined the quality and characteristics of pain experienced by patients with hip and knee OA and reported two distinct types of pain; the intermittent and constant OA pain [18]. Therefore, these groups developed the Intermittent and Constant OsteoArthritis Pain (ICOAP) measurement tool to assess the hip and knee OA pain [19]. The ICOAP questionnaire is an 11-item tool that measures hip and knee “constant pain” and “pain that comes and goes” and their impact on quality of life in terms of mood and sleep disturbance. The ICOAP scale showed adequate psychometric properties regarding inter-item correlation, content and construct validity, internal consistency and test-retest reliability [19].

The ICOAP questionnaire has been cross-culturally adapted to different languages other than the original English version [20–25]. Recently, it was cross-culturally adapted into the Arabic language [26]. However, the published Arabic version suffered significant limitations [27]. The issues noticed are mainly concerned with the correctness of the translation used in the Arabic version of the ICOAP questionnaire. The authors did not appropriately translate the ICOAP questionnaire. They used Arabic terms and item structure that do not correctly infer comparable meaning. These inconsistencies would prevent reaching equivalence between the English and Arabic versions of the ICOAP questionnaire. Accordingly, the content validity of the Arabic version would be significantly deficient, which renders it inequivalent to the original ICOAP and, consequently, invalid and inappropriate for assessing OA pain in the Arab population in its current state. Additionally, responsiveness was not reported for the Arabic ICOAP, which is an important psychometric property to assess the tool’s sensitivity to changes occurring in the measured outcomes [28].

Therefore, the purposes for the current study were to appropriately translate and cross-culturally adapt the ICOAP measurement tool into Arabic and assess the psychometric properties of the culturally adapted Arabic version (ICOAP-Ar). The examined properties were validity (content, structural and construct validity), reliability (test-retest reliability and internal consistency), and responsiveness.

## Materials and methods

### Participants

Patients with Knee OA were recruited from those referred to the outpatient physical therapy clinic of King Fahd Military Medical Complex, Saudi Arabia, and Therapy & Rehab. Center, Egypt. The study was approved by the Institutional Review Board of the King Fahd Military Medical Complex, Saudi Arabia (AFHER-IRB-2020-024). All participants received verbal and written information about the study and signed the consent form.

Eligible participants had to meet the knee OA diagnostic criteria according to the American College of Rheumatology (ACR) [7]. These include knee pain and at least three of the following additional symptoms: morning stiffness ≤ 30 min, crepitation, bone margin tenderness, bony enlargement and no palpable warmth. Patients were excluded if they: had rheumatoid arthritis, serious pathological conditions (inflammatory arthritis and malignancy), total or partial arthroplasty of the affected joint, or could not read and understand documents written in Arabic.

### Procedures

This psychometric testing study had two phases. Phase I for the translation and cross-cultural adaptation of the ICOAP measurement tool to create its equivalent Arabic version (ICOAP-Ar). Phase II was conducted to evaluate the psychometric properties of the ICOAP-Ar.

#### Phase I: translation and cross-cultural adaptation

After permission from the original developer (Dr. Gillian Hawker) was granted, and in accordance with the recommended guidelines [29], this process included the following procedures in order. Stage I, forward translation of the original ICOAP into Arabic was carried out independently by two translators; a professional translator and a musculoskeletal physical therapy consultant. Both are native Arabic speakers and bilingual in English. Stage II, based on the received translations, the principal investigator constructed a preliminary version of the ICOAP-Ar after consensus meeting with the two translators. Stage III, the preliminary ICOAP-Ar was translated back into English by two bilingual translators who are native English speakers and unfamiliar with the original ICOAP. Stage IV, a consensus panel including the translators, a language professional, a methodologist, the study investigators, a musculoskeletal clinician, and the original ICOAP scale developer (Dr. Gillian Hawker) reviewed all the translated versions and developed a prefinal version of the ICOAP-Ar. The consensus panel made decisions to achieve equivalence between the original ICOAP and the prefinal ICOAP-Ar. At this stage, the item and scale content validity was objectively assessed by calculating the Content Validity Index (CVI).

Stage V, the prefinal version of the ICOAP-Ar was pilot-tested on a sample of 30 patients with knee OA for clarity and understanding to examine its face validity. The patients were asked to complete the prefinal ICOAP-Ar. Then, the participants were interviewed to record their feedback, using written feedback reports, about the questionnaire in terms of meaning of items, clarity of instructions and ability to self-complete it, and relevance to their condition. Participants’ interview reports were reviewed by the consensus panel and the ICOAP-Ar was modified as necessary.

#### Phase II: testing the Psychometric Properties

Eligible patients with knee OA were invited to join the study and were informed about the purpose of the study. After signing a consent form, participants were instructed to complete the ICOAP-Ar. The same patients were asked to retake the ICOAP-Ar 72–96 hours later for assessing the test-retest reliability of the ICOAP-Ar. Finally, after receiving four weeks of physical therapy treatment, responsiveness of the ICOAP-Ar was assessed.

Structural validity of the ICOAP-Ar was examined using the exploratory factor analysis (EFA) to verify its dimensionality as previously recommended [19]. Then, confirmatory factor analysis (CFA) was conducted to confirm the factor structure of the ICOAP-Ar. Construct validity (convergent validity) of the ICOAP-Ar was investigated using the Hypothesis-testing method to test the relationship between the ICOAP-Ar (total and constant and intermittent pain subscales) and the pain and symptoms scores of the KOOS measurement tool. Accordingly, participants were instructed to complete the KOOS along with the ICOAP-Ar.

Different measures were calculated to assess the reliability of the ICOAP-Ar. These included the test-retest reliability, internal consistency (Cronbach’s alpha and the corrected item-total correlation), standard error of measurement (SEM), and smallest detectable change at 95% confidence interval (SDC_95_).

To evaluate responsiveness of the ICOAP-Ar, participants were reevaluated after receiving standard physical therapy treatment for a period of 4 weeks. Treatment protocols were not controlled or standardized for patients. However, general standard physical therapy treatment protocols were administered to the participants that included stretching and strengthening exercises for the lower extremity musculature, which were provided by licensed physical therapists in outpatient clinical settings. Participants were instructed to complete the ICOAP-Ar and Global Rating of Change (GRoC) scales after completing a physical therapy course. The GRoC was used as an external reference for calculation of measurement error and responsiveness. All study procedures and measures were administered and supervised by trained physical therapists.

### Instrumentation

The ICOAP is an 11-item questionnaire. Each item is rated from 0 to 4 on a 5-point Likert scale. It has two subscales: constant pain (5 items) and intermittent pain (6 items). A score is calculated for the constant (0–20) and intermittent (0–24) subscales separately and for total pain (0–44), which are further normalized to a score of 0 (no pain) -100 (extreme pain) [19].

The KOOS is a self-reported questionnaire used to subjectively assess five knee OA-relevant domains: Pain, Symptoms, Activities of Daily Living (Function), Sport and Recreation Function (Sport/Rec) and knee-related Quality of Life (QOL). It comprises 42 questions across the 5 subscales. A 5-point Likert scale is used to answer questions, and standardized answers are assigned a score from 0 (no symptoms) to 4 (extreme symptoms). Finally, a normalized score (0–100, worst to best) is calculated for each subscale [30].

The GRoC is a 15-point Likert scale used to assess the patient perceived deterioration or improvement following an intervention, ranging from − 7 (a very great deal worse) to + 7 (a very great deal better), with 0 indicating no change of condition. The GRoC includes a single question about change in health status after 4 weeks of physical therapy for knee OA [31].

### Data analysis

Descriptive statistics were calculated, and data presented using frequencies and percentages for categorical variables and mean ± standard deviation for continuous variables. The normalized scores (0-100) of the ICOAP-Ar total and subscales were used for data analysis. Statistical analysis was performed using the Statistical Package for the Social Sciences (SPSS, Chicago, IL) version 21. The level of significance was set at *P* ≤ 0.05 for all the analyses.

#### Sample size

The sample size was calculated using the PASS software (Version 20.0.2) considering the Spearman’s correlation coefficient (rho) for testing the construct validity of the ICOAP-Ar. Based on acceptable rho value of at least 0.3 (R1 in PASS), a power of 90% and p value of 0.05, a sample size of 92 participants was required.

#### Validity

The CVI, for the entire scale (S-CVI) and for each item independently (I-CVI), was used to evaluate the content validity of the ICOAP-Ar with an acceptable value of at least 0.8. Additionally, the S-CVI was calculated using the average I-CVI scores (S-CVI/Ave), and the proportion of scale items that were reported relevant by all the experts (S-CVI/UA).

The EFA was implemented using the principal component analysis with varimax rotation and eigenvalue = 1, and based on acceptable values of greater than 0.5 for the Kaiser-Meyer-Olkin (KMO) [32] and less than 0.05 for the Bartlett’s test of sphericity [33]. The goodness of model fit was examined using the CFA based on a recommended CMIN/df (degrees of freedom) value below 3, standardized Root Mean Squared Residual (SRMR) value below 0.07, and comparative fit index (CFI) value above 0.90 [34]. Factor loading above 0.3 was considered acceptable [35].

Construct validity was evaluated by calculating the rho between the ICOAP-Ar score and the relevant pain and symptoms score of the KOOS. The coefficient is classified as follows: rho = 0.3–0.7 moderate correlation, and > 0.7 strong correlation [36]. We hypothesized a priori that the ICOAP-Ar total and subscales (constant and intermittent) pain score would correlate moderately negatively with the pain and symptoms subscales of the Knee Injury and OA Outcome Score (KOOS). If five of the six predetermined hypotheses are accepted, the construct validity of ICAOP-Ar would be considered adequate [37].

#### Reliability

A Cronbach’s alpha value of ≥ 0.7, and corrected item-total correlation of ≥ 0.3 were considered acceptable [37, 38]. Test-retest reliability was assessed using the intraclass correlation coefficient (two-way mixed effects, absolute agreement, single rater/measurement). An ICC value of > 0.8, and 0.6 to 0.8 was considered as excellent and good correlation, respectively [39]. The SEM and SDC_95_ were calculated using the formula: SEM =(SD×[√(1-ICC)]), where SD was the sample’s standard deviation and SDC_95_ = SEM ×1.96 ×√2, respectively [40].

#### Responsiveness

To assess responsiveness of the ICOAP-Ar, several approaches were implemented. We compared the pre- (baseline) and post-treatment (after four weeks) ICOAP-Ar total and subscale pain scores using the Wilcoxon Signed-Ranks test. The standardized effect size (SES) and standardized response mean (SRM) were calculated to evaluate the effect size, which was interpreted as large (≥ 0.80), moderate (≥ 0.50) or small (≥ 0.20). A hypothesis-testing approach was utilized to assess the correlation between the score changes (ICOAP-Ar_change_= ICOAP-AR_final_ – ICOAP-AR_baseline_) of the ICOAP-Ar total and subscale pain scores and the GRoC. The Spearman’s correlation coefficient was calculated with the same classification as described above [41]. It was hypothesized that the score change of the ICOAP-Ar total and subscales would correlate moderately negatively with the GRoC scale.

Finally, anchor-based responsiveness of the ICOAP-Ar score was assessed adopting the GRoC score as the external anchor. Participants were categorized according to their reported GRoC scores to either improved (GRoC ≥ 3) or stable group (GRoC < 3 to >-3). Receiver operating characteristics (ROC) curve of the ICOAP-Ar final score was blotted to calculate the area under the curve (AUC). An AUC value of ≥ 0.7 was considered adequate to indicate agreement with the GRoC [37]. The cut-off score of the ICOAP-Ar scale was determined as the point on the ROC curve yielding the minimal value for (1 − sensitivity)^2^ + (1 − specificity)^2^ [42].

#### Floor and ceiling effects

The presence of floor and ceiling effects was evaluated as it compromises the responsiveness of a measurement tool. The floor and ceiling effects were considered present if more than 15% of the sample scored the lowest or highest possible score on baseline ICOAP-Ar subscales and total pain [37].

## Results

### Subjects

The demographic data of the participants are summarized in Table 1. Initially, 135 knee OA patients were approached, and 36 subjects were excluded because they did not meet the inclusion requirements. The remaining 99 patients participated in the validity assessment and only two patients dropped out during the reliability assessment (n = 97). Seventy-five patients completed the third visit (75.8%), and their data were used for responsiveness assessment.

**Table 1:** Participants’ characteristics (n = 99)

Variables	Mean (SD)	95% CI	
**Age (years)**	52.97 (9.9)	51.0–54.9	
**Stature (cm)**	160.58 (7.5)	159.1–162.1	
**Body mass (kg)**	83.54 (14.7)	80.6–86.5	
**BMI (kg/cm** ^**2)**^	32.26 (5.7)	31.1–33.4	
**Knee OA Duration (Months)**	19.85 (25.3)	14.8–24.9	
		**Frequency**	**%**
**Sex**	Men	27	27.3
	Women	72	72.7
**Level of education**	Secondary	55	55.6
	Bachelor’s degree	40	40.4
	Master’s degree	4	4
	PhD	0	0
**Affected knee**	Right knee	15	15.2
	Left knee	13	13.1
	Both knees	71	71.7
**Kellgren-Lawrence (K-L) grade**	Grade 0	0	0
	Grade 1	26	26.3
	Grade 2	43	43.4
	Grade 3	25	25.3
	Grade 4	5	5.1
**GRoC (n = 75)**	Improved	41	54.7
	Stable	34	45.3
	**Mean (SD)**	**95% CI**	
**ICOAP-Ar_Total visit 1 (n = 99)**	55.76 (20.3)	51.7–59.8	
** ICOAP-Ar_Constant visit 1 (n = 99)**	54.75 (25.4)	49.7–59.8	
** ICOAP-Ar_Intermittant visit 1 (n = 99)**	56.61 (18.7)	52.9–60.3	
**ICOAP-Ar_Total visit 2 (n = 97)**	50.66 (20.1)	46.6–54.7	
** ICOAP-Ar_Constant visit 2 (n = 97)**	49.51 (24.4)	44.6–54.4	
** ICOAP-Ar_Intermittant visit 2 (n = 97)**	51.63 (19.0)	47.8–55.5	
**ICOAP-Ar_Total visit 3 (n = 75)**	44.79 (21.1)	40.2–49.9	
** ICOAP-Ar_Constant visit 3 (n = 75)**	43.33 (24.5)	38.0–49.3	
** ICOAP-Ar_Intermittant visit 3 (n = 75)**	46.00 (20.8)	41.4–51.0	
**KOOS Symptoms (n = 99)**	58.4 (20.1)	54.4–62.5	
**KOOS Pain (n = 99)**	50.5 (19.2)	46.7–54.3	

SD: standard deviation, CI: Confidence Interval, n: number of participants, BMI: body mass index, ICOAP: Intermittent and Constant Osteoarthritis Pain Questionnaire KOOS: Knee Injury and Osteoarthritis Outcome Score, GRoC: Global Rating of Change

### Translation and cross-cultural adaptation

Few differences were identified between the original and backward-translated versions of the ICOAP. Upon meeting with the author of the ICOAP scale, the panel of experts had consensus on most of the scale items. The main concerns were related to the translation of the words “frustrated or annoyed” and “upset or worried” in item 4 and 5, respectively. These terms could have nearly similar meanings when translated into Arabic. This was also reported by the participant’s feedback during pilot testing of the prefinal version of the ICOPA-Ar. Accordingly, the panel agreed after elaboration from the author to translate “frustrated or annoyed” to “إحباط أو إنزعاج” and “upset or worried” to “الضيق أو القلق”. Other than that, the participants did not report any difficulty regarding the clarity or understanding of the ICOAP-Ar during pretesting with a measured CVI between 0.97 and 1 for all the items.

### Validity

#### Content validity

The ICOAP-Ar was very clear and easily understood by both the panel of experts and participants with an excellent I-CVI for all the items (I-CVI = 1). Similarly, the S-CVI, S-CVI/Ave and S-CVI/UA all had a perfect value of 1 (Table 2).

**Table 2:** Evaluation of I-CVIs with expert’s agreement, scales’ items

Items Experts’ rating of relevance									
	E1	E2	E3	E4	Experts in agreement	I-CVI	UA	P_c_	K^*^
**1.**	4	4	4	4	4	1	1	0.063	1
**2.**	4	4	4	4	4	1	1	0.063	1
**3.**	3	4	3	4	4	1	1	0.063	1
**4.**	4	4	3	4	4	1	1	0.063	1
**5.**	4	4	4	4	4	1	1	0.063	1
**6.**	4	4	4	4	4	1	1	0.063	1
**7.**	3	4	3	4	4	1	1	0.063	1
**8.**	4	4	4	4	4	1	1	0.063	1
**9.**	4	4	4	4	4	1	1	0.063	1
**10.**	4	4	4	4	4	1	1	0.063	1
**11.**	4	4	4	4	4	1	1	0.063	1
**Proportion relevance**	1	1	1	1	S-CVI/Ave = 1		S-CVI/UA = 1		

I-CVI = item-level content validity index; UA = Universal Agreement; P_c_ = probability of a chance computed by formula: P_c_ = [N/A(N - A)]*0.5^ N^ where N = number of experts and A = Number agreeing on good relevance; K* = modified kappa coefficient k* = (I-CVI - P_c_)/(1 - P_c_), ^a^ As stated by (Cicchetti & Sparrow, 1981);(Fleiss, 1981) about the criteria for K*, 0.40 to 0.59 = fair, 0.60 to 0.74 = good, and > 0.74 excellent

#### Structural validity

**Fig. 1:**
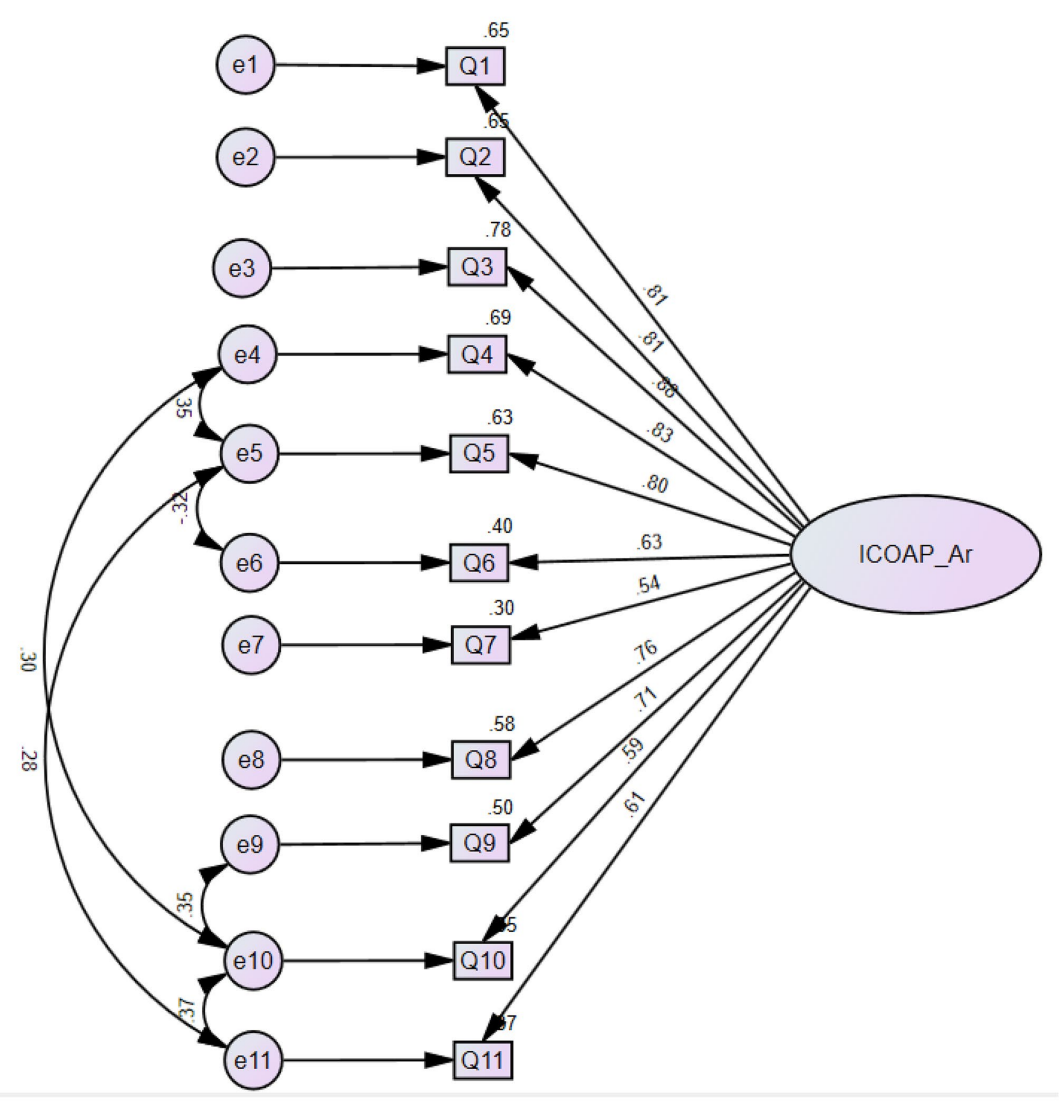
Path diagram showing factor structure of the ICOAP-Ar

The EFA showed suitability for factor analysis with KMO = 0.88 and acceptable Bartlett’s index (p < 0.001). The analysis yielded two factors that accounted for 67.8% of the variance. However, all the items loaded across the two factors. Items 1,2,3,6,7, and 8 loaded more on the first factor (loading range = 0.6–0.85), while items 4,5,10, and 11 loaded more on the second factor (loading range = 0.67–0.86). Item 9 loaded equally across factors (0.54–0.58). Therefore, we proceeded with the CFA considering a one factor model. The CFA revealed an acceptable factor loading for all the items (Fig. 1) and good fit indexes (CMIN/df = 2.6, SRMR = 0.06, and CFI = 0.92).

#### Construct validity

The results revealed that all the predefined hypotheses were confirmed. The ICOAP-Ar total and its constant and intermittent pain domains had a strong (rho between − 0.71 and − 0.76, p < 0.001) and moderate (rho between − 0.57 and − 0.68, p < 0.001) negative correlation with KOOS pain and symptoms domains, respectively. The ICOAP-Ar constant pain domain had the lowest correlation with the KOOS pain and symptoms domains, while the ICOAP-Ar total had the highest correlation with the KOOS pain domain (Table 3).

**Table 3:** Correlations between ICOAP-Ar and KOOS for construct validity

ICOAP-Ar domains	Instrument for correlation	Correlation coefficient (rho)	Hpotheses confirmed?
**ICOAP-Ar Total**	**KOOS - Symptoms domain**	-0.64*	Yes
**ICOAP_Ar Constant pain domain**		-0.57*	Yes
**ICOAP_Ar Intermittent pain domain**		-0.68*	Yes
**ICOAP-Ar Total**	**KOOS - Pain domain**	-0.76*	Yes
**ICOAP_Ar Constant pain domain**		-0.71*	Yes
**ICOAP_Ar Intermittent pain domain**		-0.74*	Yes

* *P* value is significant at ˂0.001

### Reliability

The ICOP-Ar total (α = 0.93) and its domains showed excellent internal consistency. They also had excellent test-retest reliability (ICC = 0.98 − 0.92). The SEM for the ICOAP-Ar total and its constant and intermittent pain domains was 6.1, 7.1, and 6.3, respectively. The SDC_95_ ranged between 16.8 for the ICOAP-Ar total and 19.7 for its constant pain domain (Table 4).

**Table 4:** Internal consistency, test–retest reliability (ICC), Standard Error of Measurement (SEM), Minimal Detectable Change (MDC95), and floor and ceiling effects for the ICOAP-Ar (total and domains)

Variable	Internal Consistency(α)	Test re-test reliability (95% CI)	SEM	MDC95	*Floor effect %	*Ceiling effect %
**ICOAP-Ar Total**	0.93	0.91 (0.79–0.95)	6.1	16.8	0.0	0.0
**ICOAP_Ar Constant pain domain**	0.92	0.92 (0.84–0.96)	7.1	19.7	7.1	1.0
**ICOAP_Ar Intermittent pain domain**	0.86	0.89 (0.76–0.94)	6.3	17.5	0.0	0.0

α: Cronbach alpha, ICC: Intra-class correlation; CI: confidence interval. *n = 99. Data for the SEM and MDC95 are normalized to scores 0-100

The corrected item-total correlations were acceptable for the ICOAP-Ar items. The corrected item-total correlation coefficient ranged between 0.53 and 0.82 for the total scale. For the scale domains, it ranged between 0.55 and 0.77, and 0.75 and 0.87 for the intermittent and constant pain domains, respectively (Table 5).

**Table 5:** Corrected item-total correlation (n = 99)

ICOAP items	Corrected item-total coefficients*	Corrected item-total coefficients†	Cronbach’s α if item deleted*	Cronbach’s α if item deleted†
Constant pain subscale				
1. How intense has your constant knee pain been?	0.747	0.726	0.913	0.921
2. How much has your constant knee pain affected your sleep?	0.748	0.745	0.913	0.920
3. How much has your constant knee pain affected your overall quality of life?	0.866	0.822	0.889	0.916
4. How frustrated or annoyed have you been by your constant knee pain?	0.837	0.813	0.895	0.917
5. How upset or worried have you been by your constant knee pain?	0.786	0.758	0.906	0.920
Intermittent pain subscale				
6. How intense has your most severe knee pain that comes and goes been?	0.610	0.600	0.845	0.926
7. How frequent has this knee pain that comes and goes occurred?	0.552	0.528	0.854	0.929
8. How much has your knee pain that comes and goes affected your sleep?	0.686	0.746	0.832	0.920
9. How much has your knee pain that comes and goes affected your overall quality of life?	0.768	0.731	0.816	0.921
10. How frustrated or annoyed have you been by your knee pain that comes and goes?	0.654	0.635	0.837	0.925
11. How upset or worried have you been by your knee pain that comes and goes?	0.653	0.656	0.838	0.924

* Obtained for ICOAP constant and intermittent pain subscales

† Obtained for ICOAP total pain

### Responsiveness

**Fig. 2:**
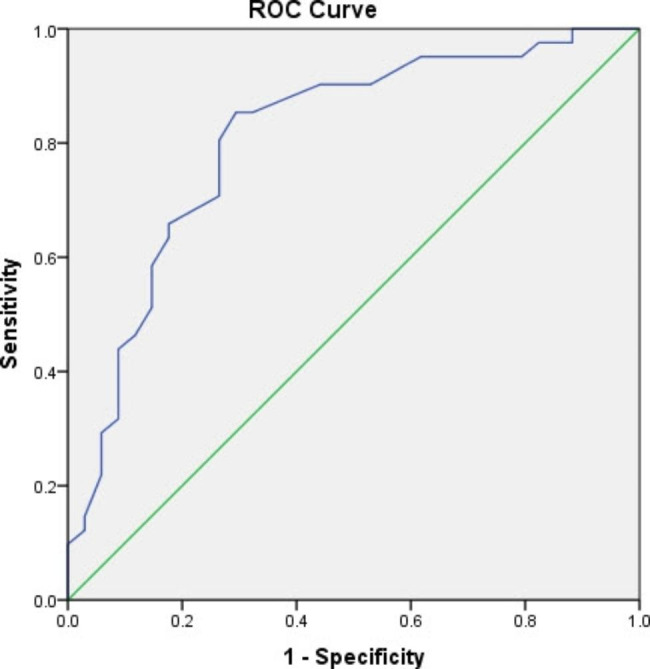
Receiver operating characteristic curve for the ICOAP-Ar (n = 75)

Forty-one (54.7%) patients reported improvement according to their GRoC score. There was a significant difference between the pre- and post-treatment ICOAP-Ar total and subscale scores (*P* < 0.001). The mean score change for the entire sample ranged between 11.9 and − 12.9. The SES (0.51–0.65) and SRM (0.86–0.99) values for the ICOAP-Ar total and subscales were moderate and large, respectively. Additionally, there was a significant negative moderate correlation (rho between − 0.44 and − 0.48, p < 0.001) between the ICOAP-Ar total and subscale change score and the GRoC (Table 6), which confirms the predefined hypothesis. Furthermore, the ICOAP-Ar showed adequate accuracy with an AUC of 0.81 (95% confidence interval = 0.71–0.91). The optimal cutoff point was 51.1 to differentiate between patients who improved and those who were stable at a sensitivity and specificity of 0.85 and 0.71, respectively (Fig. 2).

**Table 6:** Responsiveness of the ICOAP-Ar total and subscale pain (n = 75)

Variable	Pre-treatment Mean ± SD	Post-treatment Mean ± SD	Change Mean ± SD	SES	SRM	Correlation with GRoC	Hpotheses confirmed?
**ICOAP-Ar Total**	**57.2 ± 19.7**	**44.8 ± 21.1**	-12.4 ± 12.5	0.63	0.99	-0.48	Yes
**ICOAP_Ar Constant paindomain**	**56.2 ± 25.1**	**43.3 ± 24.6**	-12.9 ± 14.2	0.51	0.91	-0.44	Yes
**ICOAP_Ar Intermittent pain domain**	**57.9 ± 18.2**	**46.0 ± 20.8**	-11.9 ± 13.6	0.65	0.86	-0.44	Yes

Change = post-treatment score - pre-treatment score; improvement if change < 0

Bald values indicate significant difference comparing pre- and post-treatment scores (*P* < 0.001)

SES = |mean (change) ÷ SD (pre-treatment)|

SRM = |mean (change) ÷ SD (change)|

### Floor/Ceiling effect

No floor or ceiling effects were present (Table 4).

## Discussion

The current study cross-culturally adapted the ICOAP scale into Arabic following the international recommendations [29, 43] and examined its psychometric properties in patients with knee OA. The results showed that the ICOAP-Ar was acceptable and easily comprehended by the participants. This was corroborated by the perfect CVI value (I-CVI and S-CVI = 1) obtained from both the expert panel and participants, which is higher than that reported for the Chinese version (CVI = 0.8–1) [20]. The ICOAP-Ar also demonstrated acceptable validity, reliability, and responsiveness, which is in accordance with the previous studies [20–25].

The structural validity of the ICOAP-Ar was examined using EFA and CFA. The EFA confirmed the dimensionality of ICOAP-Ar showing the scale to have a single construct of pain. This is in accordance with the previously reported factorability of the original ICOAP. Hawker et al. (2008) reported that EFA of the ICOAP scale revealed a single pain construct suggesting sufficient homogeneity between the 11 scale items [19]. This was further confirmed by the CFA findings that showed acceptable model fit parameters with good factor loading. Since CFA is unique to the current study, we cannot compare our findings to the original or any of the previously translated versions of the ICOAP scale.

Our predictions regarding the construct validity of the ICOAP-Ar were all confirmed. The ICOAP-Ar total and subscales scores had strong (rho= -0.71 to -0.76) to moderate (rho= -0.57 to -0.68) negative correlations with the KOOS pain and symptoms subscales, respectively. This finding is in agreement with previous studies validating the Portuguese (rho= -0.61 to -0.81) [23], Persian (rho= -0.5 to -0.7) [22], and Chinese (rho= -0.65 to -0.680) [44] versions of the ICOAP. The correlation coefficient values are also comparable with those reported by other studies that used the Western Ontario and McMaster Universities Osteoarthritis Index (WOMAC) instead of the KOOS [20, 21, 44].

It is important to note that the ICOAP-Ar (total and subscales) correlation coefficients were higher with the KOOS pain domain than those calculated with the KOOS symptoms domain. This interesting finding was in line with the previous studies that validated the different versions of the ICOAP scale. It was consistently observed in studies that used the KOOS pain and symptoms subscales to examine the construct validity of the ICOAP scale [22, 23], as well as other studies that used the WOMAC pain and function subscales or the health survey (SF36 or SF12, physical component) for the same purpose [20, 25, 45]. This further confirms the divergent validity of the ICOAP-Ar as appropriate for assessing the construct of pain.

The internal consistency of the ICOAP-Ar total and subscales was excellent (Cronbach’s alpha = 0.86 to 0.93), which indicates the consistency and homogeneity of the scale items. This corresponds well with the Cronbach’s alpha values reported for the original (Cronbach’s alpha = 0.93) [19] and different language versions (Cronbach’s alpha = 0.82 to 0.97) of the ICOAP [20–23, 25, 44]. Further confirmation of the ICOAP-Ar item consistency and lack of redundancy is the acceptable corrected item-total correlation values for its total and subscales scores (ranged from 0.53 to 0.87). These correlation coefficient values are also in agreement with those reported for other language versions for the ICOAP [20, 22, 23].

The ICOAP-Ar total (ICC = 0.91) and its constant (ICC = 0.92) and intermittent (ICC = 0.89) pain subscales showed excellent test-retest reliability reflecting the questionnaire’s good reproducibility. The obtained values are slightly higher than those reported for the original version (ICC = 0.85) [19], but comparable to other language versions (ICC = 0.88–0.96) [20–23, 44]. Data analysis revealed that the ICOAP-Ar had SEM values of about 6–7 points for its total and subscale pain scores. This, accordingly, resulted in a MDC_95_ value of 16.8 points for the ICOAP-Ar total score. This means that an ICOAP-Ar score change of at least 16.8 points is required to be interpreted as a real within-subject change of knee OA pain [46].

The SEM/MDC_95_ values (3.7/10.3 points) of the ICOAP total pain were reported for the traditional Chinese version (tChICOAP) only [20]. However, for comparative purposes, we could easily calculate the SEM/MDC_95_ values for the other language versions from the published data. The SEM/MDC_95_ values were 6.7/18.5 and 6.9/19.1 points for the Persian and Portuguese ICOAP total pain, respectively [22, 23]. It is clear that the SEM/MDC_95_ values for the ICOAP-Ar total pain are highly comparable to those for the other language versions, except the tChICOAP. This could be attributed to the sample criteria and baseline tChICOAP total pain score. The baseline tChICOAP total pain score (34.52) is less than that for the ICOAP-Ar (55.76). Accordingly, one could logically speculate limited score variability for the tChICOAP compared with the ICOAP-Ar upon retest, which was represented by the higher test-retest reliability for the tChICOAP (ICC2,1 = 0.96) compared with the ICOAP-Ar (ICC2,1 = 0.91) total pain score.

Our findings provided evidence supporting the accuracy and sensitivity to change of the ICAOP-Ar after physical therapy treatment for knee OA. The ICOAP-Ar total and subscale pain scores were significantly reduced after the four-week treatment period revealing a moderate effect size (SES = 0.51–0.65), but large SRM (0.86–0.99). Responsiveness of the original ICAOP scale was previously examined, and the findings showed variable low (SES = 0.46–0.54), moderate (SES = 0.5–0.88), and large (SES = 0.93–1.71) effect size for pharmacological treatment, intra-articular injection, and joint replacement surgery, respectively [44, 45, 47–50].

Responsiveness of the ICOAP to physical therapy treatment was assessed for the Portuguese version [41]. Compared to the ICOAP-Ar, the Portuguese ICOAP has higher SES (0.83–1.42) and SRM (1.33–1.50) values after physical therapy intervention for knee OA. The higher effect size could be attributed to differences of the sample criteria and data recording approach between the two studies. The participants’ mean knee OA duration for the Portuguese ICOAP responsiveness study was 10.1 years, while it was 1.7 years for our ICAOP-Ar study. The shorter duration of knee OA could have led to less response to treatment in the current study compared with the Portuguese ICOAP one. This assumption is further corroborated by the nearly similar SES value for the constant pain subscale of the ICOAP-Ar (0.51) and the Portuguese ICOAP (0.51) for the subgroup of patients with less than 5 years duration of knee OA. Additionally, the ICOAP-Ar data were self-reported, while the Portuguese ICOAP data were interviewer-administered. The different approaches of data collection process could likely have contributed to the lower SES and SRM values for the ICOAP-Ar due to the absence of willingness to please the therapist influence or presence of a misunderstanding bias [48].

The predefined hypothesis regarding the correlation between the score changes of the ICOAP-Ar total and subscale pain and the GRoC were established showing a moderate negative correlation (rho= -0.44 to -0.48), which is consistent with the findings of the Portuguese version (rho= -0.56 to -0.64) [41]. The accuracy of the ICAOP-Ar was established with adequate AUC value (0.81) to discriminate between patients who improved with decreased knee pain after treatment and those who did not. The AUC value for the ICOAP was previously reported for the Chinese version [44] after total knee replacement (AUC = 0.92), and the Greek version [45] after intra-articular injection treatment (AUC = 0.76). A cut-off score of 51.1 was determined for the ICOAP-Ar, which is an important indicator for clinicians as it indicates that patients with an ICOAP-Ar total score of ≥ 51 points are expected to improve, and their knee pain becomes less after physical therapy treatment. Finally, and in agreement with other versions of the ICOAP, the floor and ceiling effects were not detected for the ICOAP-Ar total and subscale pain scores. These findings support the notion that the ICOAP-Ar can be used to longitudinally assess knee pain changes with acceptable accuracy.

Some limitations of this study should be addressed. Unfortunately, we were not able to validate the ICOAP-Ar scale for the hip OA patients as we could not recruit enough participants to perform reliable data analysis. Our sample may not be representative since it had a short knee OA duration. However, the impact knee OA duration may have on the psychometric properties of the ICOAP-Ar is expected to be minimal. We did not examine the responsiveness characteristics of the ICOAP-Ar in patients with knee OA receiving treatments other than the physical therapy, which we believe is important to provide comprehensive data regarding the validation of the ICOAP-Ar scale.

## Conclusions

The ICOAP scale was successfully cross-culturally adapted into Arabic following international guidelines. The ICOAP-Ar total and subscale is a valid and reliable tool to assess knee OA pain. It has also demonstrated adequate accuracy and sensitivity to knee pain change in response to physical therapy intervention. An ICOAP-Ar total score of 51.1 points could be used as a cut-off to discriminate the response of patients with knee OA to physical therapy treatment.

## Electronic supplementary material

Below is the link to the electronic supplementary material.

Supplementary Material 1

## Data Availability

The datasets for the current study are available from the corresponding author on reasonable request.

## Abbreviations

OA: Osteoarthritis
ICOAP: Intermittent and Constant Osteoarthritis Pain
Ar: Arabic
AUC: Area Under the Curve
GRoC: Global Rating of Change
tChICOAP: traditional Chinese ICOAP
ICC: Intraclass Correlation Coefficient
KOOS: Knee Injury and OA Outcome Score
ACR: American College of Rheumatology
CVI: Content Validity Index
KOOS: Knee Injury and Osteoarthritis Outcome Score
SPSS: Statistical Package for the Social Sciences
SEM: Standard Error of Measurement
SDC: Smallest Detectable Change
SES: Standardized Effect Size
SRM: Standardized Response Mean
ROC: Receiver Operating Characteristics.
