# Supplementary material for: Arabic version of the intermittent and constant osteoarthritis pain questionnaire (ICOAP-Ar): translation, cross-cultural adaptation, and measurement properties
Source: BMC Musculoskelet Disord. 2023 Jun 13;24:481. doi: 10.1186/s12891-023-06492-w (PMC10262478; doi:10.1186/s12891-023-06492-w)
Supplement: Supplementary file 1 — Supplementary Material 1 [file 12891_2023_6492_MOESM1_ESM.pdf]

مقياس ألم خشونة المفاصل المتقطع و المستمر : النسخة الخاصة بمفصل الركبة

أخبرنا الناس بأنهم يعانون من أنواع مختلفة من الألم (بما في ذلك الوجع أو عدم الارتياح) في الركبة. وللحصول على فهم أفضل لآلام الركبة التي قد تعاني منها، نود أن نطرح عليك بعض الأسئلة عن أي ألم مستمر (ألم تشعر به طوال الوقت)، وبشكل منفصل عن أي ألم قد شعرت به أحياناً أقل، وهو الألم المتقطع (الألم الذي يأتي ويذهب).
الأسئلة التالية للاستفسار عن الألم الذي عانيت منه في مفصل الركبة خلال الأسبوع الماضي. يرجى الإجابة على جميع الأسئلة.

أ) الألم المستمر

لكل سؤال من الأسئلة التالية، الرجاء اختيار الإجابة التي تصف بشكل أفضل متوسط *ألم الركبة المستمر* لديك في الأسبوع الماضي:

1. في الأسبوع الماضي، ما مدى شدة ألم الركبة المستمر؟

0 ☐	1 ☐	2 ☐	3 ☐	4 ☐
على الإطلاق/ لا يوجد ألم ركبة مستمر	خفيف	متوسط	شديد	إلى أقصى حد

2. في الأسبوع الماضي، ما مدى تأثير ألم الركبة المستمر على نومك؟

0 ☐	1 ☐	2 ☐	3 ☐	4 ☐
على الإطلاق/ لا يوجد ألم ركبة مستمر	خفيف	متوسط	شديد	إلى أقصى حد

3. في الأسبوع الماضي، ما مدى تأثير ألم الركبة المستمر على جودة حياتك بشكل عام؟

0 ☐	1 ☐	2 ☐	3 ☐	4 ☐
على الإطلاق/ لا يوجد ألم ركبة مستمر	خفيف	متوسط	شديد	إلى أقصى حد

4. في الأسبوع الماضي، ما مدى شعورك بالإحباط أو الانزعاج من ألم الركبة المستمر؟

0 ☐	1 ☐	2 ☐	3 ☐	4 ☐
على الإطلاق/ لا يوجد ألم ركبة مستمر	خفيف	متوسط	شديد	إلى أقصى حد

5. في الأسبوع الماضي، ما مدى شعورك بالضيق أو القلق من ألم الركبة المستمر؟

0 ☐	1 ☐	2 ☐	3 ☐	4 ☐
على الإطلاق/ لا يوجد ألم ركبة مستمر	خفيف	متوسط	شديد	إلى أقصى حد

ب) الألم المتقطع

لكل سؤال من الأسئلة التالية، الرجاء اختيار الإجابة التي تصف بشكل أفضل متوسط*__ألم الركبة المتقطع__* لديك في الأسبوع الماضي:

6. في الأسبوع الماضي، ما مدى شدة __ألم الركبة المتقطع__ الأكثر حدةلديك؟

☐0	☐1	☐2	☐3	☐4
على الإطلاق/<br>لا يوجد ألم ركبة متقطع	خفيف	متوسط	شديد	إلى أقصى حد

7. في الأسبوع الماضي، ما مدى تكرار حدوث __ألم الركبة المتقطع__؟

☐0	☐1	☐2	☐3	☐4
أبداً/<br>لا يوجد ألم ركبة متقطع	نادراً	أحياناً	غالباً	في أغلب الأحيان

8. في الأسبوع الماضي، ما مدى تأثير__ألم الركبة المتقطع__ على نومك؟

☐0	☐1	☐2	☐3	☐4
على الإطلاق/<br>لا يوجد ألم ركبة متقطع	خفيف	متوسط	شديد	إلى أقصى حد

9. في الأسبوع الماضي، ما مدى تأثير __ألم الركبة المتقطع__ على جودة حياتك بشكل عام؟

☐0	☐1	☐2	☐3	☐4
على الإطلاق/<br>لا يوجد ألم ركبة متقطع	خفيف	متوسط	شديد	إلى أقصى حد

10. في الأسبوع الماضي، ما مدى شعورك بالإحباط أو الانزعاج من __ألم الركبة المتقطع__؟

☐0	☐1	☐2	☐3	☐4
على الإطلاق/<br>لا يوجد ألم ركبة متقطع	خفيف	متوسط	شديد	إلى أقصى حد

11. في الأسبوع الماضي، ما مدى شعورك بالضيق أو القلق من __ألم الركبة المتقطع__؟

☐0	☐1	☐2	☐3	☐4
على الإطلاق/<br>لا يوجد ألم ركبة متقطع	خفيف	متوسط	شديد	إلى أقصى حد

شكراً لكم،،،
